# Postmarketing safety surveillance of dexamethasone intravitreal implant in the treatment of visual impairment due to diabetic macular edema in India

**DOI:** 10.1186/s12886-020-01630-7

**Published:** 2020-10-09

**Authors:** Unnikrishnan Nair, Vishali Gupta, Mohita Sharma, Shrinivas Joshi, Aditya Sudhalkar, Undraa Altangerel, Yan Bai, for the India Ozurdex Postmarketing Surveillance Study Group, Manisha Agarwal, Divya Balakrishnan, Alay Banker, Nishikant Borse, Vishali Gupta, Shrinivas Joshi, Manoj Khatri, Jyotsna Myneni, Manish Nagpal, Unnikrishnan Nair, R. Rajesh, Vishal R. Raval, Rajarami Reddy, Sudhir Salhotra, Manoj Saswade, Mohita Sharma, Indu Singh, Anshuman Sinha, Aditya Sudhalkar

**Affiliations:** 1 Chaithanya Eye Hospital & Research Institute, Trivandrum, Kerala 695004 India; 2 Postgraduate Institute of Medical Education & Research, Advanced Eye Center, Chandigarh, India; 3 Tirupati Eye Centre, Noida, Gautam Budh Nagar, Uttar Pradesh, India; 4 M.M. Joshi Eye Institute, Hosur Hubli, Karnataka India; 5 Raghudeep Eye Hospital, Ahmedabad, Gujarat India; 6 Global Patient Safety and Epidemiology (GPSE), Allergan, an AbbVie Company, Irvine, CA USA

**Keywords:** Corticosteroid, Dexamethasone, Diabetic macular edema, Postmarketing, Safety profile

## Abstract

**Background:**

Diabetic macular edema (DME) is the most common cause of vision loss in diabetic patients. As India has the second largest population of diabetic patients worldwide, availability of various treatment options for DME is essential. This postmarketing surveillance study was conducted to fulfill a commitment to the Regulatory Authority of India to examine the safety of dexamethasone intravitreal (DEX) implant over 1 year in Indian patients with DME receiving ≥1 DEX implant for DME-related visual impairment in clinical practice.

**Methods:**

This observational, prospective, non-interventional study enrolled patients aged ≥18 years scheduled to receive DEX implant for DME-related visual impairment. Baseline demographics, medical history, date of last DEX implant injection, detailed information about adverse events (AEs), AEs of special interest (AESIs), serious AEs (SAEs), and adverse drug reactions (ADRs) reported during postinjection visits and investigator telephone calls were collected. Primary outcome measures were treatment-emergent AE (TEAE), AESI, SAE, and ADR occurrences.

**Results:**

Of the enrolled patients (19 sites throughout India; *n* = 250), 84 had received DEX implant previously; mean (standard deviation; SD) duration between prior and study entry dose was 199.4 (156.0) days, and 91 (36.4%) had ≥1 prior ophthalmic condition. Over a mean of 182.6 (88.6) follow-up days (min–max: 0–364 days), 22 TEAEs were reported by 7 (2.8%) patients, 6 of whom had previously received DEX. AESIs of increased IOP (*n* = 3, 6 events) and glaucoma (*n* = 1, 1 event) were considered non-serious, of mild/moderate severity, and related to DEX treatment. Eyelid ptosis was reported in 1 patient (1 event). Nonocular AEs included cardiac AEs (*n* = 3, 4 events), pyrexia (*n* = 1, 2 events), and dyspnea (*n* = 1, 2 events). Three (1.2%) patients had 12 serious AEs; most were cardiac disorders; all were unrelated to DEX treatment. Two (0.8%) deaths were considered unrelated to treatment.

**Conclusions:**

Based on voluntary reporting of adverse events in this surveillance study, DEX implant for treatment of DME-related visual impairment in the Indian population demonstrated a favorable safety profile with few treatment-related TEAEs (none were considered serious) during the 1-year follow-up. These data supplement previous findings and confirm the safety of DEX implant in this population during usual clinical practice.

**Trial registration:**

World Health Organization Clinical Trials Registry: CTRI/2017/04/008396. Registered 24 April 2017.

## Background

India has the second largest population of patients with diabetes, a global epidemic, with projected increases from 65.1 million in 2013 to 109 million affected individuals by 2035 [1]. The increase in the incidence of diabetes infers an increase in the prevalence of diabetic macular edema (DME), which has been reported to be approximately 7.5% (age-standardized) in patients with diabetes and is the most common cause of vision loss in these patients [2, 3].

Inflammation plays a prominent role in the pathogenesis of DME, as evidenced by the expression of various inflammatory factors such as vascular endothelial growth factor (VEGF), intercellular adhesion molecule-1, interleukin-6, and monocyte chemotactic protein-1 [4] following breakdown of the blood retinal barrier, as well as leukostasis and endothelial tight junction protein alterations [5]. Patients with DME present with capillary leakage, fluid accumulation, and retinal thickening subsequent to blood retinal barrier breakdown [6]. Notwithstanding some instances of spontaneous recovery, DME is typically a chronic condition, with approximately 25% of affected patients experiencing moderate visual loss (≥15 letters on the Early Treatment Diabetic Retinopathy Study chart) in the absence of treatment within a 3-year period [6].

The current approach to treatment subdivides DME according to involvement at the center of the macula, with a greater risk of visual loss and need for treatment when the center is involved. Photocoagulation, previously the mainstay of therapy, is now recommended for non-center-involving DME but is limited by possible adverse events (AEs) leading to visual field loss [7–9]. Based on a number of well-designed clinical trials, intravitreal VEGF antagonists have been adopted as the primary therapy for patients with center-involved DME [10, 11]. The demonstrated ability of intravitreal corticosteroids to inhibit expression of inflammatory mediators such as VEGF and intercellular adhesion molecule-1 [12], inhibit leukostasis [13], and augment the barrier function of vascular endothelial tight junctions [14], positions these agents as an effective therapeutic alternative in patients with DME.

Dexamethasone intravitreal implant (DEX; Ozurdex, Allergan, an AbbVie company, North Chicago, IL) contains 0.7 mg (700 μg) dexamethasone in a solid biodegradable sustained-release drug delivery system and is indicated for the treatment of macular edema following branch and central retinal vein occlusion, noninfectious posterior segment uveitis, and DME [15, 16]. In the two multicenter, double-masked, randomized, pivotal phase 3 trials, a single injection of DEX demonstrated a significantly accelerated ability to achieve ≥15 letter (3-line) improvement in best-corrected visual acuity in patients with DME compared with the sham at 30, 60, and 90 days after a single injection (all *p* < 0.05). This effect was detected within the first 2 months after implantation in approximately 10 to 15% of DEX-treated patients and persisted approximately 1 to 3 months [15]. The safety profile of DEX in these studies was better than that reported in studies of other intraocular steroids when used in patients with DME, with no unexpected AEs and excellent systemic safety.

The present postmarketing safety surveillance study was conducted to evaluate the real-life clinical experience with DEX implant in India, per post-approval commitment, as a supplement to established safety data. The safety profile of DEX was assessed by actively identifying and evaluating the occurrence of AEs and serious adverse events (SAEs) over a 1-year period during usual clinical practice in adult Indian patients who received at least one intravitreal DEX injection for the treatment of visual impairment due to DME.

## Methods

### Study design

This observational, prospective, non-interventional, postmarketing surveillance program was conducted from December 2016 to December 2017 at 19 sites across India. Data regarding DEX administration were collected during usual clinical practice, at the discretion of the treating physician. Study sites and prescribing physicians were selected by the study sponsor based on the geography of the site to approximate DME patients treated in the real world clinical setting in India. The study was designed and performed in accordance with Good Clinical Practice and adhered to the tenets of the Declaration of Helsinki. Institutional ethics committee (IEC) approval was obtained at 8 of the 19 investigator sites where there was a provision; the remaining investigator sites were clinic settings and did not have the provision of ethics approval per the country-specific requirements [17, 18]. Signed written informed consent was obtained from all subjects before initiation of data collection. The study is registered in the World Health Organization Clinical Trials Registry with the identifier CTRI/2017/04/008396.

### Patient selection

The study subjects comprised adult patients aged ≥18 years who were scheduled to receive at least one intravitreal DEX injection for the treatment of visual impairment due to DME. Subjects were excluded from the study if they presented with one of the following conditions: ocular or periocular infections, glaucoma, a torn or ruptured posterior lens capsule, or hypersensitivity. Eligible patients were invited by the investigator to enroll in the study at the time of presentation for a routine clinic visit.

### Treatment

DEX injection was administered at the discretion of the physician/investigator at the enrollment visit or the subsequent visit. The study sponsor did not provide study medication.

### Outcome measures

The primary outcome measures were the occurrence of serious AEs, adverse events of special interest (AESIs), AEs, and adverse drug reactions (ADRs). A serious AE was considered any AE that occurred at any dose that resulted in death, a life-threatening AE, inpatient hospitalization or prolongation of an existing hospitalization, persistent or significant disability/incapacity, or a congenital anomaly/birth defect. The AESIs were glaucoma or increased intraocular pressure (IOP). An AE was considered any untoward medical occurrence in a patient who was administered the pharmaceutical product and did not necessarily have a causal relationship with the treatment. The severity of an AE was established by clinical determination as follows: mild (awareness of sign or symptom, but easily tolerated), moderate (discomfort enough to cause interference with usual acuity), or severe (incapacitating with inability to work or do usual activity). An ADR was considered a response to a drug which is noxious and unintended, and which occurs at doses normally used in humans for prophylaxis, diagnosis, or therapy of diseases, or for modification of physiological function. AEs were determined and reported as such by the investigator and categorized based on clinical judgement.

### Assessments

At the enrollment visit (visit 1), a pre-designed clinical report form was used to collect patient demographics, relevant medical and ophthalmic history, concomitant medications, date of the last DEX injection, if applicable, date of the present DEX injection, and detailed information about SAEs, AESIs, AEs, and ADRs following DEX treatment. AEs were collected once informed consent was obtained, regardless of whether the patient had been administered study drug.

Post-injection evaluations were not mandated. All follow-ups (clinic visits/telephone contact) were scheduled at the discretion of the physician/investigator, and similarly, results of follow-up evaluations were reported at the physician’s/investigator’s discretion only if they deemed them as AEs. During the follow-up visits (or telephone contact), the physician/investigator collected information about SAEs, AESIs, AEs, and ADRs (including dates, if available) on the form, and recorded newly prescribed/administered medications (regardless of their association with an AE). Each patient interview started with simple open-ended questions and questions designed to collect information regarding the specific outcomes. If the patient was seen by a non-study physician, the study physician made every effort to follow-up to facilitate accurate reporting.

Data collected for AEs consisted of the term used to describe the event, onset and resolution dates, seriousness, severity (mild, moderate, or severe), outcome, treatment, relationship of the event to intravitreal DEX injection as determined by the study physician, the action taken in terms of DEX, and the concomitant medications administered for the AE (start date, stop date, dose, unit, and frequency). Medical records were used to collect data to determine exposure, effects, and outcomes. Other variables such as potential confounding variables and effect modifiers were also collected.

Upon the occurrence of an SAE, the site informed the governing IEC of the SAE as required by the IEC. Furthermore, as this was a postmarketing surveillance study, reporting of an AE or SAE to the governing health authority followed the applicable regulations for a marketed product (ie, all cases involving serious unexpected AEs were reported to the licensing authority by the study sponsor within 15 days of initial receipt of the information, and other non-serious AEs were reported through a Periodic Safety Update Report that followed the prescribed periodicity).

All events were recorded and sent to the sponsor’s global safety team using the designated postmarketing form: SAEs within 24 h of awareness of the event and AEs within 10 calendar days. SAEs were also reported to the governing IEC.

### Statistical methods

The analysis population included all enrolled patients who met eligibility criteria and were administered at least one DEX injection. All data collected on the clinical report form were transferred to a clinical database and then imported into SAS (Cary, NC, USA) for further analysis. Medical history, current medical conditions, and SAEs, AEs, and ADRs were coded using the *Medical Dictionary for Regulatory Activities version 20.1* nomenclature and presented by primary system organ class and preferred term. The frequency of treatment-emergent SAEs, AESIs, SAEs, and ADRs during the study period was summarized in one of two ways: (1) the number and percentage of patients reporting each event, counting those who reported multiple episodes of the same event only once, divided by the number of patients who were treated with DEX during the study period; and (2) the number and percentage of each event, counting multiple episodes of the same event separately, divided by the number of patients who were treated with DEX during the study period. The safety profile was further evaluated by patients with and without prior DEX treatment.

Continuous data are presented as the mean (95% confidence interval [CI] or standard deviation [SD]) and median (minimum and maximum). Categorical data are presented as percentages. The number of non-missing records was determined. Missing values as a consequence of patients who did not return to follow-up visits were not replaced and were treated as censored. Thus, no imputation for missing data was performed.

Because this was an observational, prospective, non-interventional study without a comparison group, no formal sample-size calculation was performed. However, based on the enrollment potential, approximately 250 patients from 20 sites across India were planned to be enrolled as a representative sample.

## Results

### Patient demographics and clinical characteristics

A total of 250 eligible patients were enrolled in the study, received at least one intravitreal DEX injection, and were included in the analysis. Baseline patient demographics and characteristics are presented in Table 1. The patient population was predominantly male and the mean age was 60 years. The majority of patients were reported to have a diagnosis of diabetes mellitus type 2 (*n* = 188 [75.2%]) and 60 (24%) patients reported having diabetes mellitus but did not specify the type. A total of 57 (22.8%) patients reported a history of diabetic retinal edema, 22 (8.8%) diabetic retinopathy, and 18 (7.2%) a history of cataract (9 patients reported cataract surgery). No patient reported a history of glaucoma.

**Table 1:** Patient baseline demographics and clinical characteristics

Demographic/characteristic	All enrolled patients (*N* = 250)
Age, y, mean (SD)	60.2 (9.4)
Male, *n* (%)	169 (67.6)
Type 2 diabetes,^a^ *n* (%)	188 (75.2)
≥1 Relevant ophthalmic condition,^b^ *n* (%)	91 (36.4)
Time since diagnosis, y, mean (SD)	1.7 (2.6)
Treated eye,^c^ *n*	91
Right only, *n* (%)	17 (18.7)
Left only, *n* (%)	13 (14.3)
Both, *n* (%)	61 (67.0)
Prior medications for DME, *n*	
DEX	84
Dexamethasone (systemic)	6
Bevacizumab	5
Ranibizumab	4
Treated eye, *n*	84
Right only, *n* (%)	35 (41.7)
Left only, *n* (%)	45 (53.6)
Both, *n* (%)	4 (4.8)
Concomitant medications for diabetes, *n* (%)	
Metformin	114 (45.6)
Glimepiride	76 (30.4)
Concomitant medications for cardiovascular conditions, *n* (%)	
Telmisartan	55 (22.0)
Atorvastatin	32 (12.8)
Blood and blood-forming agents	44 (17.6)
Last prior DEX dose to study entry dose, d	
*n*	80
Mean (SD)	199.4 (156.0)
Min, max	0, 891

*DEX* dexamethasone intravitreal implant; *DME* diabetic macular edema; *SD* standard deviation

^a^Type was not specified in 60 (24%) patients; ^b^“Relevant ophthalmic conditions” in medical history includes all ophthalmic conditions; ^c^Five patients reported condition as “not treated” despite all patients reporting one (right/left) or both eyes being treated

Most patients were DME treatment-naïve at baseline, with 34% of patients previously treated with intravitreal DEX injection, 2.4% with systemic dexamethasone, 2.0% with intravitreal bevacizumab, and 1.6% with intravitreal ranibizumab (Table 1). The latter three therapies were stopped prior to study enrollment. The mean (SD) duration between the prior dose of DEX and the dose administered at study entry was 199.4 (156.0) days. Among those previously treated with DEX, the proportions of right (41.7%) and left eyes (53.6%) previously treated were fairly balanced; 4 patients (4.8%) had received DEX in both eyes.

At least one relevant ophthalmic condition was reported in 91 (36.4%) patients at enrollment, with a mean (SD) duration since diagnosis of 1.7 (2.6) years (Table 1). Of the 91 patients with an ophthalmic condition, all 91 had received treatment in one or both eyes, 61 had received treatment in both eyes, and 5 reported the condition as “not treated.” The most common nonocular medical conditions other than diabetes mellitus were hypertension (*n* = 154 [61.6%]) and hypothyroidism (*n* = 13), coronary artery disease, and myocardial infarction (*n* = 7 for each). The majority of patients were receiving oral antidiabetic agents and medications for cardiovascular disease (Table 1).

### Serious adverse events

In all, 12 treatment-emergent SAEs were reported in 3 (1.2%) patients during the study, all of which were nonocular and most frequently classified in the primary system organ class of cardiac disorders (angina pectoris, unstable angina, cardiac arrest, and myocardial infarction; Table 2). Additional SAEs included pyrexia, dyspnea, acute kidney injury, fluid overload (in one patient) and acute hepatic failure, septic shock (in a second patient). Eleven of the 12 events were deemed serious as they required in-patient hospitalization or prolongation of existing hospitalization and the other because the event resulted in death. Of note, 2 of the 3 patients (0.8%) with SAEs died during the study. A 63-year-old male patient died of a severe myocardial infarction 32 days after the last DEX injection. Another male patient aged 56 years old, who was diagnosed with severe acute hepatic failure 101 days after the last DEX injection, experienced septic shock and cardiac arrest, and died 19 days later. Both deaths were considered to be unrelated to the study treatment.

**Table 2:** Treatment-emergent serious adverse events (all events)

MedDRA system organ class/preferred term	Number of patients^a^ (%)	Number of events^b^ (%)
All	3 (1.2)	12 (4.8)
Cardiac disorders	3 (1.2)	4 (1.6)
Angina pectoris	1 (0.4)	1 (0.4)
Angina unstable	1 (0.4)	1 (0.4)
Cardiac arrest	1 (0.4)	1 (0.4)
Myocardial infarction	1 (0.4)	1 (0.4)
General disorders and administration site conditions	1 (0.4)	2 (0.8)
Pyrexia	1 (0.4)	2 (0.8)
Respiratory, thoracic and mediastinal disorders	1 (0.4)	2 (0.8)
Dyspnea	1 (0.4)	2 (0.8)
Hepatobiliary disorders	1 (0.4)	1 (0.4)
Acute hepatic failure	1 (0.4)	1 (0.4)
Infections and infestations	1 (0.4)	1 (0.4)
Septic shock	1 (0.4)	1 (0.4)
Metabolism and nutrition disorders	1 (0.4)	1 (0.4)
Fluid overload	1 (0.4)	1 (0.4)
Renal and urinary disorders	1 (0.4)	1 (0.4)
Acute kidney injury	1 (0.4)	1 (0.4)

*MedDRA* Medical Dictionary for Regulatory Activities; *SAE* serious adverse event

^a^Number of patients experiencing each SAE; patients who report multiple episodes of the same AE are counted only once. ^b^Number of SAEs reported; each reported event is counted, including multiple episodes of the same event by the same patient

### Treatment-emergent adverse events

Over a mean (SD) follow-up of 182.6 (88.6) days (min–max: 0–364 days), 22 TEAEs were reported by 7 (2.8%) patients (Table 3). The majority of the patients with a reported TEAE had previously received DEX treatment (*n* = 6 of 7 patients). The AESIs, which were reported in 3 (1.2%) patients, were increased IOP (6 events in 3 patients) and glaucoma (1 event in 1 patient). Increased IOP, the most common AE reported in the study, was reported in 3 patients (6 events). Five of the occurrences of increased IOP were considered to be moderate and 1 was considered to be mild in severity. Glaucoma was also reported in 1 of these 3 patients (1 event); however, IOP increase and glaucoma could not be differentiated as AEs, as AEs were captured as reported by the treating physicians in the patient report form. All AESIs were considered to be non-serious, treatment-related, and were categorized as ADRs.

**Table 3:** Treatment-emergent adverse events (> 1 reported event within a system organ class)

MedDRA system organ class/preferred term	Number of patients^a^ (%)	Number of events^b^ (%)
All^c^	7 (2.8)	22 (8.8)
Investigations	3 (1.2)	6 (2.4)
Intraocular pressure increased	3 (1.2)	6 (2.4)
Cardiac disorders	3 (1.2)	4 (1.6)
Angina pectoris	1 (0.4)	1 (0.4)
Angina unstable	1 (0.4)	1 (0.4)
Cardiac arrest	1 (0.4)	1 (0.4)
Myocardial infarction	1 (0.4)	1 (0.4)
Eye disorders	2 (0.8)	2 (0.8)
Eyelid ptosis	1 (0.4)	1 (0.4)
Glaucoma	1 (0.4)	1 (0.4)
General disorders and administration site conditions	1 (0.4)	2 (0.8)
Pyrexia	1 (0.4)	2 (0.8)
Renal and urinary disorders	1 (0.4)	2 (0.8)
Acute kidney injury	1 (0.4)	1 (0.4)
End-stage renal disease	1 (0.4)	1 (0.4)
Respiratory, thoracic, and mediastinal disorders	1 (0.4)	2 (0.8)
Dyspnea	1 (0.4)	2 (0.8)

*AE* adverse event; *MedDRA* Medical Dictionary for Regulatory Activities

^a^Number of patients experiencing each AE; patients who report multiple episodes of the same AE are counted only once. ^b^Number of AEs reported; each reported event is counted, including multiple episodes of the same event by the same patient. ^c^Category encompasses both ocular and nonocular treatment-emergent AE

Among nonocular TEAEs, a total of 4 cardiac AEs were reported by 3 (1.2%) patients: angina pectoris, unstable angina, cardiac arrest, and myocardial infarction, all of which were classified as SAEs (described earlier) (Tables 2 and 3). Two events of pyrexia, also considered SAEs, were reported in 1 (0.4%) patient and 2 events of dyspnea were reported in 1 (0.4%) patient. Two events of renal and urinary disorders were reported in 1 patient, comprising acute kidney injury (categorized as serious) and end-stage renal disease. All other AEs and SAEs occurred no more than 1 time each.

The TEAE of IOP increase was reported in 2 (0.8%) patients with prior DEX treatment and was considered to be moderate and related to the study treatment; the treating physician reported both IOP increase and glaucoma on the same date in 1 of these 2 patients, and this patient’s glaucoma was ongoing at the time of study completion. In the patient naïve to prior DEX, the TEAE of IOP increase was considered to be mild and related to the study treatment.

**Fig. 1:**
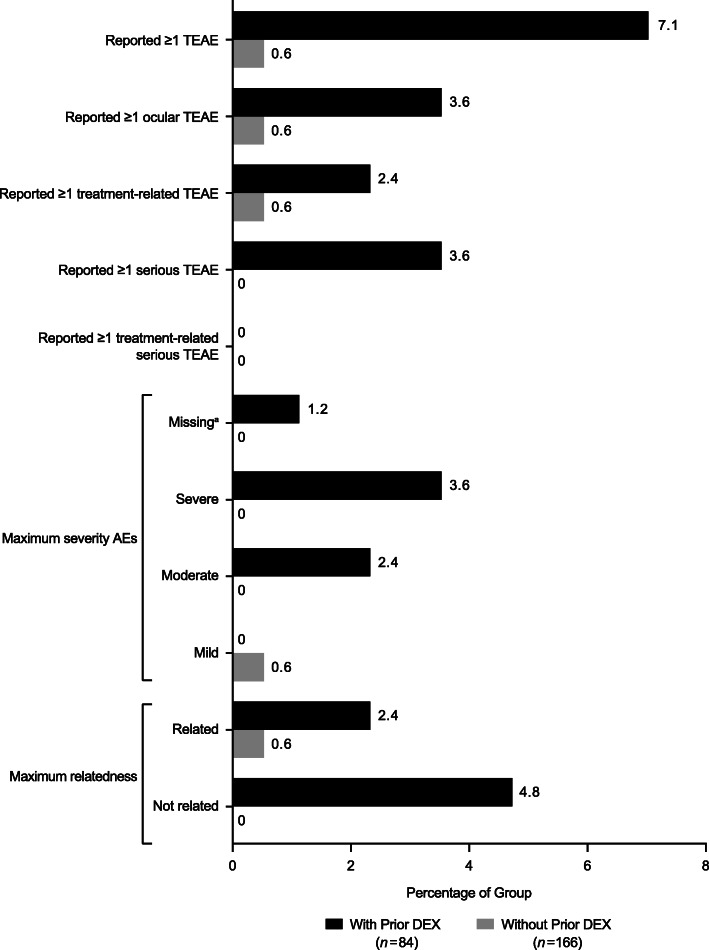
Summary of treatment-emergent adverse events (TEAEs) by prior dexamethasone intravitreal implant (DEX) treatment status. ^a^The severity of an AE (eyelid ptosis) was noted to be “mild” as clarified by a data clarification form. However, this information was not reported in the database

An overall summary of TEAEs by prior DEX treatment, their severity, and whether they were deemed by the investigator to be treatment-related is provided in Fig. 1.

### IOP-lowering therapy

IOP-lowering medications were prescribed for 18 (7.2%) patients, including the 2 patients who had AE reports of moderate IOP increases. These agents included fixed combinations dorzolamide hydrochloride/timolol maleate and brimonidine tartrate/timolol maleate, and bimatoprost, travoprost, brinzolamide, and acetazolamide. In 13 of the 18 patients, the IOP-lowering therapy was specifically used as prophylaxis or postoperative care and/or initiated on the date of DEX injection. In the other 5 patients, IOP-lowering therapy was initiated shortly prior to the date of DEX injection. Of the 13 patients, 11 (84.6%) had no reports of IOP increase as an AE over the course of the study. The remaining 2 patients were mentioned previously with moderate IOP increases: although they initiated IOP-lowering therapy on the date of DEX injection, they subsequently had incidents of IOP increase that were reported as AEs. One patient discontinued the initial IOP-lowering therapy after 7 days and then received another regimen for an AE of moderate IOP increase 92 days later. The second patient had 4 incidents of IOP increase, with events ranging in duration from 2 to 13 days; this patient received 1 IOP-lowering medication at DEX injection, followed by 2 additional medications at the onset of the first AE report of increased IOP and 1 additional medication on the second onset date. No patient receiving DEX underwent glaucoma incisional surgery for increases in IOP. Notably, the majority of the patients who received IOP-lowering medications (13 of 18) had a history of prior DEX injection, but whether they also had a history of IOP increases after those injections was not captured in this study.

## Discussion

In this real-world study examining the occurrence of AEs in adult patients in India who received at least one intravitreal DEX injection for DME, the proportion of patients with a reported AE was relatively low, given the observational nature of the study and voluntary reporting of AEs. TEAEs related to the intravitreal DEX implant, which were reported in only 1.2% of the treated patient population, were IOP increase and glaucoma. All other AEs were considered unrelated to the implant and were reported of mild or moderate severity. The repeated treatment with DEX in previously treated patients did not appear to introduce any new safety concerns.

Subsequent to its initial approvals for the treatment of retinal vein occlusion-associated macular edema and noninfectious posterior segment uveitis, DEX was approved in the United States for the treatment of DME and in the European Union for adults with visual impairment due to DME who are pseudophakic or who are considered insufficiently responsive to or unsuitable for noncorticosteroid therapy. The DEX registration study for DME (MEAD study) comprised patients who were treated with DEX and followed for up to 3 years, with at least 6-month intervals between treatments [15]. The results of that seminal study showed an acceptable long-term safety profile of the intravitreal DEX implant, with improvements over other ocular corticosteroids in patients with DME, and no unexpected AEs or evidence of incremental systemic AEs or increased risk for thromboembolic events following repeated treatment. Consistent results were later obtained in a real-life, bicentric, retrospective study examining the safety and efficacy of intravitreal DEX injection in 128 eyes of 89 patients with DME (RELDEX study), the findings of which demonstrated favorable 3-year efficacy and safety outcomes in real-life practice [19].

In agreement with previous studies addressing the long-term use of intravitreal DEX in clinical practice for retinal vein occlusion-associated macular edema [16, 20] and cystoid macular edema associated with quiescent non-infectious posterior segment uveitis [21], the most commonly reported AESIs in the present analysis were increased IOP and glaucoma. In fact, these AESIs were reported in a markedly smaller proportion of the population in the present study than in previous analyses; however, this discrepancy could potentially be explained by differences in the design of the studies, including the shorter duration of the present investigation, which, due to the longer duration often observed for the development of cataract with corticosteroid use, may have minimized the incidence of AEs by excluding those that develop beyond 1 year post-injection.

The reported use of IOP-lowering medications as prophylaxis and postoperative care in this study agree with some precedents in the literature: use of IOP-lowering prophylaxis to prevent IOP increases with administration of intravitreal corticosteroids [22, 23], including with DEX injection [24, 25], have previously been reported and suggest that prophylaxis can effectively limit the occurrence of IOP increases with these treatments. The finding that prophylactic IOP-lowering medications were used in a real-world setting may be of interest in clinical scenarios in which IOP increase with DEX injection is a concern.

Limitations of the present study include its observational design and the fact that it was solely conducted to collect and assess safety data. The single-arm design also precluded comparative assessments of causality. In addition, the short duration of the study may have led to an exclusion of TEAEs that developed over a longer course of follow-up. Other limitations were the lack of hypothesis testing to assess statistical significance and an inability to assess whether the participating clinics had recorded all concomitant medications and evaluation results uniformly, particularly given the fact that data reporting was fully physician-dependent. Additionally, in the absence of mandated study visits, missing data were not replaced and were treated as censored; thus, no imputation for missing data was performed. However, it must be underscored that the information gleaned from this study is considered solely as a supplement to established safety data for DEX implant and that this study was performed in part to fulfill a regulatory commitment.

## Conclusions

Application of the intravitreal DEX implant for the treatment of visual impairment due to DME in the Indian population during usual clinical practice was found to have a favorable safety profile with few voluntarily reported treatment-related TEAEs over the 1-year follow-up period. None of these treatment-related TEAEs were considered serious. The safety results of this postmarketing surveillance study supplement previously reported findings for intravitreal DEX injection for the treatment of visual impairment due to DME, confirming its safety in the Indian population.

## Data Availability

AbbVie Inc. will share de-identified patient-level data and study-level data including protocols and clinical study reports for phase 2, 3 or 4 trials completed after 2008 that are registered to ClinicalTrials.gov or EudraCT, have received regulatory approval in the United States and/or the European Union in a given indication and the primary manuscript from the trial has been published. To request access to the data, the researcher must sign a data use agreement and any shared data are to be used for non-commercial purposes. More information can be found on http://www.allerganclinicaltrials.com/.

## Abbreviations

AE: Adverse event
AESI: Adverse event of special interest
ADR: Adverse drug reaction
DEX: Dexamethasone intravitreal implant
DME: Diabetic macular edema
IEC: Institutional ethics committee
IOP: Intraocular pressure
MedDRA: Medical Dictionary for Regulatory Activities
SAE: Serious adverse event
SD: Standard deviation
TEAE: Treatment-emergent adverse event
VEGF: Vascular endothelial growth factor
